# Development of the Global Network for Women’s and Children’s Health Research’s socioeconomic status index for use in the network’s sites in low and lower middle-income countries

**DOI:** 10.1186/s12978-020-01034-2

**Published:** 2020-12-17

**Authors:** Archana B. Patel, Carla M. Bann, Ana L. Garces, Nancy F. Krebs, Adrien Lokangaka, Antoinette Tshefu, Carl L. Bose, Sarah Saleem, Robert L. Goldenberg, Shivaprasad S. Goudar, Richard J. Derman, Elwyn Chomba, Waldemar A. Carlo, Fabian Esamai, Edward A. Liechty, Marion Koso-Thomas, Elizabeth M. McClure, Patricia L. Hibberd

**Affiliations:** 1 Lata Medical Research Foundation, Nagpur, India; 2 Datta Meghe Institute of Medical Sciences, Wardha, India; 3 RTI International, Research Triangle Park, NC USA; 4 INCAP, Guatemala City, Guatemala; 5 University of Colorado, Denver, CO USA; 6 Kinshasa School of Public Health, Kinshasa, Democratic Republic of the Congo; 7 University of North Carolina At Chapel Hill, Chapel Hill, NC USA; 8 Aga Khan University, Karachi, Pakistan; 9 Department of Obstetrics and Gynecology, Columbia University, New York, NY USA; 10 KLE Academy of Higher Education and Research’s J N Medical College, Belagavi, India; 11 Thomas Jefferson University, Philadelphia, PA USA; 12 University of Zambia, Lusaka, Zambia; 13 University of Alabama At Birmingham, Birmingham, AL USA; 14 Moi University, Eldoret, Kenya; 15 Indiana University, Indianapolis, IN USA; 16 Eunice Kennedy Shriver National Institute of Child Health and Human Development, Rockville, MD USA; 17 Department of Global Health, Boston University School of Public Health, Boston, MA USA

**Keywords:** Socioeconomic status, Disparities, Determinants of health, Global health, Lower and middle income countries (LMIC), Global Network for Women’ and Children’s Health Research

## Abstract

**Background:**

Socioeconomic status (SES) is an important determinant of health globally and an important explanatory variable to assess causality in epidemiological research. The 10th Sustainable Development Goal is to reduce disparities in SES that impact health outcomes globally. It is easier to study SES in high-income countries because household income is representative of the SES. However, it is well recognized that income is poorly reported in low- and middle- income countries (LMIC) and is an unreliable indicator of SES. Therefore, there is a need for a robust index that will help to discriminate the SES of rural households in a pooled dataset from LMIC.

**Methods:**

The study was nested in the population-based Maternal and Neonatal Health Registry of the Global Network for Women’s and Children’s Health Research which has 7 rural sites in 6 Asian, sub-Saharan African and Central American countries. Pregnant women enrolling in the Registry were asked questions about items such as housing conditions and household assets. The characteristics of the candidate items were evaluated using confirmatory factor analyses and item response theory analyses. Based on the results of these analyses, a final set of items were selected for the SES index.

**Results:**

Using data from 49,536 households of pregnant women, we reduced the data collected to a 10-item index. The 10 items were feasible to administer, covered the SES continuum and had good internal reliability and validity. We developed a sum score-based Item Response Theory scoring algorithm which is easy to compute and is highly correlated with scores based on response patterns (r = 0.97), suggesting minimal loss of information with the simplified approach. Scores varied significantly by site (p < 0.001). African sites had lower mean SES scores than the Asian and Central American sites. The SES index demonstrated good internal consistency reliability (Cronbach’s alpha = 0.81). Higher SES scores were significantly associated with formal education, more education, having received antenatal care, and facility delivery (p < 0.001).

**Conclusions:**

While measuring SES in LMIC is challenging, we have developed a Global Network Socioeconomic Status Index which may be useful for comparisons of SES within and between locations. Next steps include understanding how the index is associated with maternal, perinatal and neonatal mortality.

*Trial Registration* NCT01073475

**Plain English summary:**

Socioeconomic status (SES) is an important determinant of health globally, and improving SES is important to reduce disparities in health outcomes. It is easier to study SES in high-income countries because it can be measured by income and what income is spent on, but this concept does not translate easily to low and middle income countries. We developed a questionnaire that includes 10 items to determine SES in low-resource settings that was added to an ongoing Maternal and Neonatal Health Registry that is funded by the National Institutes of Child Health and Human Development’s Global Network. The Registry includes sites that collect outcomes of pregnancies in women and their babies in rural areas in 6 countries in South Asia, sub-Saharan Africa and Central America. The Registry is population based and tracks women from early in pregnancy to day 42 post-partum. The questionnaire is easy to administer and has good reliability and validity. Next steps include understanding how the index is associated with maternal, fetal and neonatal mortality.

## Background

Socioeconomic status (SES) of individuals or families is a composite measure of an individual’s and community’s access to resources, that accounts for economic and social position in relation to others [1]. SES is an important determinant of health in high and middle- and low-income countries (LMIC) across a wide range of health conditions and diseases. In general, the lower an individual’s socioeconomic position the worse their health. The importance of SES is highlighted in one of the United Nations’ Sustainable Development Goals, to reduce income inequality (Goal 10), which has increased by 11 percent in developing countries in recent years [2]. The role of SES in maternal and child health outcomes in LMIC has therefore become a focus in the 2000s [3–5].

One of the reasons for the delays in recognition of the role of SES in health outcomes in LMIC has been determining the optimal way to measure SES. Income and consumption expenditures are widely used in high income countries to measure SES [6] but these concepts do not translate easily to many LMIC settings, particularly rural settings where the economy is often informal and difficult to track, and expenditures on health care may not be accurately recorded [7]. Alternative approaches include using household assets as a proxy for income [8].

Several SES indices have been developed; however, each has limitations for use in predicting child outcomes in LMIC. The Demographic and Health Surveys (DHS), which have been conducted in more than 90 countries since 1994 [9], are one of the most commonly referenced sources of information on SES based on asset ownership as a proxy for wealth. Wealth is considered as an underlying unobserved variable. DHS developed country-specific indices which categorize the household’s economic status in five wealth categories and allow for comparisons of wealth among individuals within the country. However, these indices were not designed for comparisons between countries. A comparative wealth index score that allows for measurement of variation in wealth among individuals within a country while also allowing for differentiation in wealth across countries is needed for a globally pooled data set [10].

To address multi-country comparisons, the United Nations Development Programme introduced the Multidimensional Poverty Index in 2010 as a new multi-country approach to understand how people experience poverty in multiple and simultaneous ways. An indicator of acute multidimensional deprivation, it identifies a state of poverty through three equally weighted dimensions: education (number of years of schooling), health (child mortality, nutritional status), and standard of living (household attributes/asset ownership) [11]. However, including health measures, such as child mortality, in the index itself restricts its suitability for predicting health-related outcomes. In addition, the index does not evaluate SES on a continuum score but rather categorizes households as poor, severe poverty and vulnerable. Therefore, it is not able to discriminate across a range of SES, limiting its sensitivity.

Some recent studies (such as the 8 Country MAL-ED (Etiology, Risk Factors and Interactions of Enteric Infections and Malnutrition and the Consequences for Child Health and Development) study [12] and the single country SHINE (Sanitation, Hygiene, Infant Nutrition, Efficacy) Trial [13] have developed new SES indicators based on the DHS and the UN index, respectively, that appear to be valid and robust. However, both of these studies designed the SES measures to optimize prediction of a specific outcome (e.g., child’s height-for-age Z-score), limiting their generalizability. Another measure, the International Wealth Index, was developed to allow for comparisons across countries [14]; however, it uses the same set of items and scoring algorithm for all countries and therefore, cannot account for country-level differences in item functioning.

Since 2009, The *Eunice Kennedy Shriver* National Institute of Child Health and Human Development’s (NICHD’s) Global Network (GN) for Women and Children’s Health Research has supported a population-based Maternal and Newborn Health Registry (MNHR) of pregnant women and their babies living in rural communities in LMIC. The MNHR has focused on documentation of maternal mortality, fetal loss after week 20 of pregnancy, accurate and timely measurement of birth weight, and early and late neonatal outcomes [15]. The GN has used the number years of maternal education as a proxy for SES since 2009 [16, 17]. In 2016, the GN revisited this approach and adapted the multipoverty index to create a simple index of SES.

The objective of this study is to use item response theory to develop and evaluate an index to assess the SES of the communities in LMICs participating GN’s MNHR which can both differentiate among participants within a country as well as permit comparisons across countries. The justification for this approach are the challenges and complexity of addressing SES in multi-country studies, including our network that is used to evaluate multiple interventions to improve maternal and neonatal mortality and to study trends of these outcomes over time.

## Methods

### Design and setting of the study

The SES study was added to the GN MNHR which collects data on a prospective cohort of pregnant women enrolled in 7 rural sites in 6 countries including 3 sites in sub-Saharan Africa, 3 sites in south Asia and 1 site in Central America. These rural study sites are in Guatemala, India (2 sites: Nagpur and Belgaum), Pakistan, Kenya, Zambia and the Democratic Republic of the Congo. Each site has included 6 to 24 distinct geographic locations (clusters). Pregnant women were recruited as early as possible during pregnancy in defined geographic catchment areas (baseline assessment) and followed at labor and delivery (birth assessment) through day 42 post-partum (outcome assessment) to obtain maternal, fetal and neonatal outcomes [15]. Pregnant women intending to deliver in the study communities were informed about the study and invited to participate in the MNHR. Those who consented were enrolled.

Starting in December 2016, all sites started to collect data on items of household conditions and assets. The candidate items from the poverty index were selected consensually by the site investigators. These items had to be applicable to their country and also be able to contribute to cross-country comparisons for a pooled data set. The data was collected during a study visit for the MNHR. These items were used to derive a measure of SES in the study population. These supplemental data were collected either during enrollment or at the day 42 post-partum assessment. Specific training materials were developed for administration of the SES questions, and all study data were subject to the GN’s standard quality control procedures [16].

### Ethical approvals

The MNHR study and questions used to devise SES were reviewed and approved at all of the involved institutions’ ethics review committees at each recruiting site and all the US based partner institutions: Kinshasa School of Public Health, Kinshasa, Democratic Republic of the Congo, University of North Carolina at Chapel Hill; University Teaching Hospital, Lusaka, Zambia; University of Alabama at Birmingham; Moi University School of Medicine, Eldoret, Kenya; Indiana University School of Medicine; The Lata Medical Research Foundation, Nagpur, Maharashtra, India; Boston University Medical Campus; KLE University’s JN Medical College, Belagavi, Karnataka, India; Thomas Jefferson University; Aga Khan University, Karachi, Pakistan; Columbia University; INCAP Guatemala City, Guatemala; University of Colorado; and RTI International. The study was registered at ClinicalTrials.gov (NCT01073475). A Data Monitoring Committee appointed by NICHD reviewed the MNHR data on at least an annual basis.

### Study participants

All pregnant women enrolled in the MNHR during the study period were enrolled in the “SES study”. There were no additional inclusion or exclusion criteria for the SES study, although study participants could refuse to answer the SES questions without compromising their participation in the MNHR.

### Items used to derive the GN socioeconomic status index

We adapted 16 items from the poverty index on housing conditions and assets owned by the participant’s household [11] to serve as the potential item pool for the SES index. Specifically housing conditions included number of people and rooms in the home, source of drinking water, sanitation facilities, type of flooring material, and type of fuel for cooking. Household assets included bicycle, motorbike, car/truck/tractor, electricity, television, refrigerator, computer, flip phone, and smart phone. All items were dichotomized with 1 indicating higher SES (i.e., household has the item) and 0 indicating lower SES (i.e., household does not have the item).

### Statistical methods

Several analyses were conducted to evaluate the performance of the candidate items and to identify the final set of items for the SES index. The percentage of respondents who reported having the item was calculated overall and by site. Confirmatory factor analyses were conducted using Mplus [18] to test the unidimensionality of the items (i.e., determining whether they cluster together into a single factor). Criteria for a good model fit included comparative fit index and Tucker-Lewis index greater than 0.95 and Root Mean Squared Error of Approximation less than 0.06 [19, 20].

After establishing unidimensionality, item response theory analyses were conducted using the IRTPRO program [21] to further examine item performanceand develop a scoring algorithm. A two-parameter logistic Item Response Theory model was fit which is appropriate for dichotomous items. Items with higher slopes are more strongly related to the underlying construct being measured by the scale (i.e., SES) and have a better ability to discriminate between respondents with high vs. low SES. Item thresholds indicate the level of SES participants would generally need before having a 50% probability of endorsing the item; higher thresholds indicate higher SES items.

Final item selection was based on a balance of statistical and content considerations. Ideal items had high factor loadings (> 0.4) and Item Response Theory slopes (> 1.0) and demonstrated variability in responses (i.e., no floor or ceiling effects). In addition, we selected items with Item Response Theory threshold parameters ranging across the SES continuum to ensure precision of measurement at both higher and lower levels of SES. To ensure content validity, we included items on both housing conditions and assets owned by the household and the final set of items was reviewed for content by in-country experts at each of the sites.

As a balance between the usability of simple sum scores and precision of Item Response Theory scores based on response patterns, we computed a total score for the SES index using a sum score to Item Response Theory expected a posteriori score conversion [22]. We then transformed these scores, so final scores on the SES index would range from 0 to 100.

To determine whether item performance varied across the sites, we also ran the confirmatory factor analyses and Item Response Theory analyses separately by site. To ensure that the final scores would permit comparisons between sites, as well as within sites, we used items that demonstrated good discrimination across the sites based on Item Response Theory slopes and factor loadings as anchor items when estimating Item Response Theory parameters for the final scoring algorithms. However, in cases where an item demonstrated poor discrimination for a particular site (slope < 0.9 or factor loading < 0.4), we estimated site-specific parameters for that item. In addition, given the very few to no participants at the DRC site who had a refrigerator or used liquefied petroleum gas /electricity as cooking fuel, we removed those items from the index for the DRC site and included two additional items (bicycle and more than one room in home) which demonstrated good discrimination for that site.

Internal consistency reliability of the index was estimated using Cronbach’s alpha. We assessed construct validity by comparing index scores for groups that would be expected to vary in terms of socioeconomic status. Specifically, we conducted analyses of variance to compare mean SES index scores among groups based on type of education (formal vs. no formal education), number of years of education (0, 1–6, 7–12, and 13 or more years), having received antenatal care (yes/no), and delivering in a hospital (yes/no). Those with more education, who received antenatal care, and delivered in a hospital are expected to have higher SES.

## Results

Between December 2016 and December 2017, 49,536 pregnant women participated in the SES study. The demographic characteristics of the women are shown in Table 1. Seventy-nine percent of the women were between the ages of 20 and 35 years. Approximately one-third of the women were nulliparous. Eighty-two percent had some formal education with 52% of the sample having 7–12 years of education. About half (49%) had received antenatal care before being enrolled in the MNHR, a number which rose to 97% by delivery. Nearly half (45%) delivered in a hospital.

**Table 1:** Demographic characteristics of the pregnant women in the study (N = 49,536)

Characteristic	N	%
Site		
Democratic Republic of Congo	6998	14
Guatemala	10,287	21
India (Belagavi)	7309	15
India (Nagpur)	7776	16
Kenya	7104	14
Pakistan	4416	9
Zambia	5646	11
Age		
< 20	7814	16
20–35	38,976	79
> 35	2714	5
Parity		
0	16,516	33
1–4	28,044	57
> 4	4951	10
Type of education		
No formal education	8857	18
Formal education	40,655	82
Years of education		
0	8857	18
1–6	11,059	22
7–12	25,907	52
13+	3686	7
Received any antenatal care		
Yes	32,420	97
No	1034	3
Delivered in a health facility		
Yes	14,557	45
No	17,731	55

The final GN SES index included ten items for each site. Eight of the items were common across all sites: finished floor, flush toilet, improved source of drinking water, electricity, television, smart phone, car, and motorbike. For the remaining two items, the index for all sites except DRC included LPG/electricity for cooking fuel and refrigerator and the index for DRC included bicycle and having more than one room in the home. The percentages of respondents with each of the final set of SES items are shown by site and overall in Table 2. As expected, given the differing wealth of the countries, there is site-level variability in terms of housing conditions and assets with participants at the India sites tending to own the most items and participants at the DRC site owning the least.

**Table 2:** Percentage of women’s households with selected global network SES index items by site

Item	All sites, (N = 49,536)	Central America	Asia			Africa		
		Guatemala, (N = 10,287)	Belagavi, India, (N = 7309)	Nagpur, India, (N = 7776)	Pakistan, (N = 4416)	Democratic Republic of Congo, (N = 6998)	Kenya, (N = 7104)	Zambia, (N = 5646)
Housing conditions								
Finished floor material	53.0	75.6	80.5	69.0	49.6	0.4	17.5	66.9
Flush toilet	26.0	42.7	32.2	59.3	25.7	0.1	0.5	6.5
LPG/electricity for cooking fuel	27.2	19.3	55.6	74.7	24.7	0.0	1.2	7.9
Improved source of drinking water	82.6	96.0	92.6	97.7	87.6	39.6	74.4	84.4
More than one room in home	81.1	95.4	96.4	95.5	58.0	52.7	62.5	92.0
Household assets								
Electricity	60.3	95.6	98.8	98.1	62.7	0.1	8.6	32.1
Television	53.2	79.3	86.6	90.6	33.3	0.4	11.2	44.3
Refrigerator	19.2	25.7	22.2	45.9	14.1	0.0	1.4	17.0
Smart phone	33.7	64.8	54.4	29.5	13.6	0.2	22.1	27.9
Car	8.3	17.8	12.7	6.5	9.2	0.1	1.2	6.4
Motorbike	34.1	23.0	68.4	73.4	53.1	4.9	15.5	1.0
Bicycle	41.4	36.9	45.7	63.4	1.7	43.2	37.1	48.2

No participants at the DRC site had LPG/electricity for cooking fuel

Due to the scarcity of the refrigerator and LPG/electricity for fuel items at the DRC site, we included two additional, lower SES items for that site (bicycle and > 1 room in home). A one-factor Confirmatory Factor Analysis model of the 10 items common across most sites fit well. All items had factor loadings greater than 0.40, further supporting the unidimensionality of the index (Table 3). Based on the model fit indices, the one-factor model also had a good fit when tested among each of the sites individually.

**Table 3:** One-factor confirmatory factor analyses of global network SES index items

Item	All sites	Central America	Asia			Africa		
		Guatemala	India (Belagavi)	India (Nagpur)	Pakistan	Democratic Republic of Congo	Kenya	Zambia
Factor loadings								
Housing conditions								
Finished floor material	0.82	0.73	0.48	0.77	0.85	0.57	0.76	0.77
Flush toilet	0.79	0.69	0.62	0.25	0.83	0.83	0.83	0.88
LPG/electricity for cooking fuel	0.85	0.71	0.67	0.74	0.89		0.86	0.86
Improved source of drinking water	0.67	0.46	0.09	0.28	0.18	0.26	0.26	0.46
More than one room in home	–	–	–	–	–	0.61	–	–
Household assets								
Electricity	0.99	0.88	0.63	0.55	0.79	0.76	0.92	0.96
Television	0.95	0.81	0.80	0.77	0.92	0.97	0.92	0.90
Refrigerator	0.85	0.77	0.82	0.88	0.92		0.90	0.96
Smart phone	0.64	0.58	0.73	0.75	0.59	0.59	0.69	0.42
Car	0.55	0.57	0.47	0.57	0.22	0.73	0.73	0.54
Motorbike	0.67	0.45	0.70	0.76	0.35	0.94	0.53	0.31
Bicycle	–	–	–	–	–	0.60	–	–
Model fit indices								
CFI	0.99	0.94	0.95	0.96	0.96	0.97	0.98	0.99
TLI	0.99	0.92	0.94	0.95	0.94	0.97	0.98	0.98
RMSEA	0.05	0.07	0.05	0.05	0.09	0.02	0.04	0.06

The items for refrigerator and LPG/electricity for fuel were excluded for the DRC site

*CFI* comparative fit index, *TLI* Tucker-Lewis index, and *RMSEA* root mean square error of approximation

**Fig. 1:**
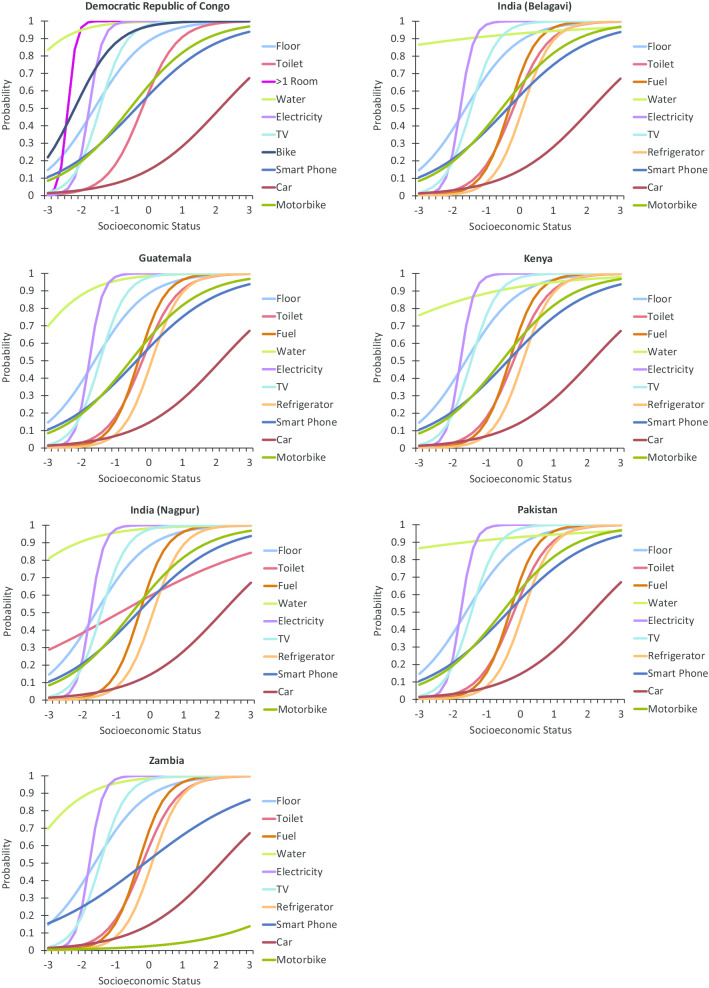
Item characteristic curves of the relationship between SES and probability of endorsing global network SES index items by site

The Item Response Theory-based item characteristic curves for each item by site are shown in Fig. 1. Overall, the items demonstrated good discrimination with steep curves, indicating they can distinguish between those with high vs. low SES. In addition, the items are spread across the SES continuum, indicating a range of threshold (*b*) parameters (i.e., point of maximal discrimination).

SES index scores were then computed using the scoring table based on the Item Response Theory parameters (Table 4). These sum score-based Item Response Theory scores were highly correlated with Item Response Theory scores based on response patterns (r = 0.97), suggesting minimal loss of information with the use of the simplified approach. Scores varied significantly by site (p < 0.001). African sites had lower mean SES scores than the Asian and South American sites: Democratic Republic of Congo (11.0), Kenya (17.1), Zambia (35.3), Pakistan (37.7), Guatemala (55.5), Belagavi, India (60.8), and Nagpur, India (62.1).

**Table 4:** Scoring table

Sum score	SES score						
	Guatemala	India (Belagavi)	India (Nagpur)	Pakistan	Democratic Republic of Congo	Kenya	Zambia
0	0.00	0.00	0.00	0.00	0.00	0.00	0.00
1	7.12	4.66	6.39	6.27	6.24	8.38	13.08
2	19.20	17.73	15.87	17.01	16.18	24.61	25.59
3	31.71	30.25	27.45	31.28	24.98	40.45	38.92
4	42.19	40.57	37.83	43.68	33.76	52.63	48.94
5	51.42	50.00	46.74	52.81	43.72	60.67	57.09
6	61.04	59.91	56.12	61.68	53.96	68.03	66.32
7	71.44	70.53	67.05	72.00	64.36	76.31	76.46
8	82.01	81.37	78.76	83.43	76.19	84.98	86.22
9	92.15	91.88	89.64	94.62	88.69	93.44	93.83
10	100.00	100.00	100.00	100.00	100.00	100.00	100.00

For all sites except Democratic Republic of Congo (DRC), sum score is the number of the following items owned by the household: finished floor material, flush toilet, LPG/electricity for cooking fuel, improved source of drinking water, electricity, television, refrigerator, smart phone, car, and motorbike. For DRC, sum score is the number of the following items owned by the household: finished floor material, flush toilet, improved source of drinking water, more than one room in home, electricity, television, refrigerator, smart phone, car, motorbike, and bicycle

The SES index demonstrated good internal consistency reliability (Cronbach’s alpha = 0.81). As shown in Table 5, comparisons of mean SES scores by education, antenatal care, and location of delivery supported the construct validity of the index. Higher SES scores were significantly associated with formal (vs. informal) education, more years of education, having received antenatal care, and delivering in a hospital (p < 0.001).

**Table 5:** Mean SES index scores by site, education, and location of delivery

Characteristic	Mean (SD)	Regression coefficient (SE)	p-value
Site			
Democratic Republic of Congo	11.01 (9.33)	− 51.11 (0.33)	< 0.001
Kenya	17.13 (18.24)	− 44.99 (0.33)	< 0.001
Zambia	35.26 (22.13)	− 26.86 (0.35)	< 0.001
Pakistan	37.67 (25.86)	− 24.44 (0.38)	< 0.001
Guatemala	55.49 (21.05)	− 6.62 (0.30)	< 0.001
India (Belagavi)	60.82 (20.72)	− 1.30 (0.33)	< 0.001
India (Nagpur)	62.12 (20.83)	Referent	
Type of education			
No formal education	27.62 (23.36)	− 17.06 (0.32)	< 0.001
Formal education	44.69 (27.99)	Referent	
Years of education			
0	27.62 (23.36)	− 40.01 (0.51)	< 0.001
1–6	34.24 (25.02)	− 33.38 (0.50)	< 0.001
7–12	45.88 (27.33)	− 21.74 (0.46)	< 0.001
13+	67.63 (25.46)	Referent	
Received any antenatal care before delivery			
Yes	42.06 (28.23)	6.37 (0.89)	< 0.001
No	35.69 (26.60)	Referent	
Delivered in hospital			
Yes	56.25 (24.53)	25.47 (0.28)	< 0.001
No	30.78 (25.38)	Referent	

Referent = reference category

## Discussion

While years of maternal education is attractive as a simple indicator of SES because it only requires one question to be answered, it has limited ability to discriminate across the range of SES [12, 13] We developed a brief, easy to score ten-item SES index using data from 7 sites in 6 LMIC based on housing conditions and household assets. The Global Network SES Index has several important characteristics. First, it has good content coverage, including items on both housing conditions and household assets. Second, using Item Response Theory, we showed that these items covered the SES continuum. Third, index demonstrated good reliability and validity. For an example of validity and as expected, index scores increased with increasing years of education and those who received antenatal care had a higher index score. The significance of the Global Network SES Index is that it is designed to obtain scores which can differentiate SES within a site using site specific scores but also permit comparison across sites by using items that performed similarly across the sites as anchor items.

In the DHS and other studies, the most commonly used statistical approach to assessment and scoring of SES indices is Principal Components Analysis [23–27]. Generally, the SES index score is computed by weighting the items according to their loadings from this analysis. The loadings provide information about how correlated the item is to the other items on the index and are similar in interpretation to loadings from factor analyses and the discrimination (i.e., slope) parameters from IRT. However, a limitation of this approach is that it does not capture additional information about the items that may be obtained using Item Response Theory. Specifically, while principal component/factor analyses assumes that an item functions equally as well across the entire continuum of SES, Item Response Theory recognizes that an item’s performance (i.e., discrimination) varies by level of SES (i.e., some items may function well for those with low SES while others may function well for those with high SES). In Item Response Theory, the location of optimal performance is captured by estimating threshold parameter(s) for each item. For this reason, we have focused on Item Response Theory to more fully evaluate the performance of each item to inform item selection and then incorporate this additional information into the computation of the index scores.

Our approach has similarities to the approach used for the MAL-ED study in which a 12-item index was developed for SES [12]. However, the assets in the MAL-ED measure of SES included 8 different assets, years of maternal education, household income and whether water and sanitation were improved. Their SES measure was designed to understand differences in stunting and therefore included different items that were related to the causal pathway to stunting such as malnutrition and enteric disease. Similarly, the SHINE Trial developed a 16-item index to measure wealth also related to the outcome of stunting, so it also included items that may not be as relevant to maternal, fetal and neonatal outcomes.

Strengths of our approach include its development in a well-established population-based Registry at 7 sites in South Asian, sub-Saharan Africa, and Central American countries that include a wide range of socioeconomic conditions both within and between countries. An additional strength included the standardized training before data collection commenced and quality control processes used by the GN to ensure that there are as few missing data as possible and that range and logic checks are applied to ensure that out of range values are correct. Our consensual approach of site investigators for selection of the candidate items in the index included Item Response Theory parameters that ensure that selected items work well across the range of SES. Our index appears valid as it discriminated between demographic characteristics that are related to SES. Finally, our 10-item index is logistically feasible to administer in a diverse range of settings and does not involve sensitive questions such as household income (that could be difficult for respondents to provide) or occupation that are less useful as an indicator of SES in LMIC countries where occupation may not vary in a rural area.

Our study had several limitations. While our study population was population based, it was not designed to be representative of the country in which our GN sites were based, so our results cannot be compared with national data that uses other measures of SES and could potentially be used to assess validity. Similarly, we cannot compare our SES with the indices developed for the MAL-ED and SHINE Trials because there are few overlapping variables We also recognize that the MAL-ED and SHINE Trials have different inclusion and exclusion criteria for study households. Finally, we accept that our GN SES Index may or may not be relevant to other studies and settings because it has not been validated beyond the GN. As with other SES indices, it may have applicability beyond our study populations but further studies are needed to address these important issues.

## Conclusion

Understanding SES as a determinant of health is increasingly recognized to be of major importance in LMIC. The overall goal of measuring SES is to identify the gaps and ultimately reduce disparities in SES globally to improve health outcomes. Measurement of SES in LMIC is generally more difficult than in high income countries because income and consumption expenditures that are widely used and established in high income countries to measure SES do not translate well to low-income settings where income is often unstable and reporting infrastructure is limited [28]. There are many limitations with the currently used approaches, particularly years of maternal education alone as a proxy for SES. Similarly, the optimal statistical approach to the development of measures of SES is not clear and there is no gold standard for comparison. We developed a 10-item index that was feasible to administer in the GN which has 7 sites in 6 south Asian, sub-Saharan African and Central American countries. The index obtains information on housing conditions and household assets, does not include sensitive questions, covers the SES continuum and has good reliability and validity. Next steps include understanding how the index is associated with maternal, fetal and neonatal mortality and other health outcomes.

## Data Availability

Study data will be available through the NICHD data and specimen hub (NDASH available at https://dash.nichd.nih.gov/).

## Abbreviations

SES: Socioeconomic status
LMIC: Low- and middle- income countries
GNSI: Global network socioeconomic status index
DHS: Demographic and Health Surveys
MAL-ED: Etiology, Risk Factors and Interactions of Enteric Infections and Malnutrition and the Consequences for Child Health and Development
SHINE: Sanitation, Hygiene, Infant Nutrition, Efficacy
NICHD’s: *Eunice Kennedy Shriver* National Institute of Child Health and Human Development’s
GN: Global Network for Women and Children’s Health Research
MNHR: Maternal and Newborn Health Registry
